# Bacterial Contamination of Clothes and Environmental Items in a Third-Level Hospital in Colombia

**DOI:** 10.1155/2012/507640

**Published:** 2012-03-26

**Authors:** J. C. Cataño, L. M. Echeverri, C. Szela

**Affiliations:** ^1^Infectious Diseases Section, Internal Medicine Department, University of Antioquia Medical School, Calle 67 # 53-108, Medellin, Colombia; ^2^Microbiology Section, San Vicente Foundation University Hospital, Calle 64 # 51D-154, Medellin, Colombia; ^3^Harvard Medical School, Medical Degree Candidate 2012, 25 Shattuck Street, Boston, MA 02115, USA

## Abstract

*Objective*. This study evaluates the bacterial contamination rate of items in the hospital setting that are in frequent contact with patients and/or physicians. By determining the bacterial species and the associated antibiotic resistance that patients are exposed to. *Methods*. Hospital-based cross-sectional surveillance study of potential bacterial reservoirs. Cultures from 30 computer keyboards, 32 curtains, 40 cell phones, 35 white coats, and 22 ties were obtained. *Setting*. The study was conducted an urban academic 650-bed teaching hospital providing tertiary care to the city of Medellin, Colombia. *Results*. In total, 235 bacterial isolates were obtained from 159 surfaces sampled. 98.7% of the surfaces grew positive bacterial cultures with some interesting resistance profiles. *Conclusion*. There are significant opportunities to reduce patient exposure to frequently pathogenic bacteria in the hospital setting; patients are likely exposed to many bacteria through direct contact with white coats, curtains, and ties. They may be exposed to additional bacterial reservoirs indirectly through the hands of clinicians, using computer keyboards and cell phones.

## 1. Background

Antibiotic-resistant bacteria are implicated in an increasing amount of hospitalized patient infections worldwide. Among patients diagnosed with an infection, antibiotic resistance is associated with an increased length of hospital stay, health care costs, and patient morbidity, and mortality. Improved hand hygiene, environmental cleaning, and isolation of patients carrying pathogenic bacteria are the main methods for tackling the problem. Despite clear evidence that hygiene improves surgical outcomes, there remains considerable controversy over whether or not contaminated environmental surfaces contribute to transmission of healthcareassociated pathogens [1–8]. The risk of nosocomial infection depends on a number of factors. These include the ability of pathogens to remain viable on a surface, the rate at which contaminated surfaces are touched by patients and healthcare workers, the context in which the patient is exposed, and the levels of contamination that result in transmission to patients. Recent studies suggest that contaminated environmental surfaces may play an important role in transmission of healthcare-associated pathogens [9–23]. Clothing including white coats appears to be contaminated in the first several hours of use [24]. Other personnel effects with frequent hand contact such as pens, stethoscopes, and cell phones may have even higher levels of contamination [25].

This study demonstrates how cloth (white coats, curtains, and ties), computer keyboards, and cell phones may act as reservoirs for bacterial pathogens that may be associated with healthcare-associated infections.

## 2. Methods

### 2.1. Setting

The study was conducted in Hospital Universitario San Vicente Fundacion, an urban academic 650-bed teaching hospital providing tertiary care to the city of Medellin, Colombia. HCWs were randomly approached during routine daily patient care, and representative surfaces were randomly sampled during typical weekdays.

### 2.2. Sample Collection and Bacteriological Analysis

Samples were randomly collected from 30 keyboards, 32 curtains, 40 cell phones, 35 white coats, and 22 ties. At the time of the study, no active investigation was being performed for a nosocomial pathogen.

Curtains were sampled in a standardized aseptic fashion. The examiner first washed his hands and then put on a sterile surgical glove then swabbed the glove along a 25 cm^2^ area on the lateral edge of the middle section of the curtain, because this is the area that HCWs most often contact with their hands when opening or closing the curtains. A hand imprint of the surgical glove was immediately printed onto a plate with blood agar for culture.

Ties and white coats were sampled in similar aseptic fashion by swabbing a sterile surgical glove along the whole cloth and then placing the glove onto a plate with blood agar for culture.

Keyboards and cell phones were sampled in a standardized aseptic fashion with sterile cotton-tipped applicators moisturized with Brain-Heart Infusion (BHI) liquid media. Then, the applicators were immediately used to inoculate BHI liquid transport media and sent directly to the laboratory for further procedures.

All liquid cultures were incubated for 24 hours at 35.5°C and then streaked on solid media culture plates, which were incubated for 48 hours at 35.5°C. 

All isolates were Gram-stained, identification of the species and antibiotic resistance was performed by a Vitek Gram-positive and Gram-negative card (*bioMérieux SA, Marcy l'Etoile, France*) according to the manufacturer's recommendations.

## 3. Results

In June 2011, a total of 159 samples were collected from 30 keyboards, 32 curtains, 40 cell phones, 35 white coats, and 22 ties. From all surfaces, 98.7% had bacterial contamination, and a total of 235 unique colonies were obtained.

### 3.1. Keyboards

From 30 keyboards sampled, a total of 39 isolations were obtained, from those, 22 (56.4%) were considered potentially clinically relevant (Table 1), highlighting bacteria as *Escherichia hermannii, *Methicillin-resistant *S. epidermidis* (MRSE),* Enterococcus faecalis, Pantoea agglomerans, *and Vancomycin-resistant *Enterococcus faecium*.

### 3.2. Curtains

From 32 curtains sampled, a total of 59 isolations were obtained, from those, 47 (79.6%) were considered potentially clinically relevant (Table 2), highlighting bacteria as Methicillin-resistant *S. haemolyticus *(MRSH), Methicillin-resistant *S. cohnii *(MRSC), MRSE, Methicillin-resistant *S. saprophyticus *(MRSS)*, Moraxella sp., Acinetobacter ursingii, *AMP-C producer *Pseudomonas oryzihabitans, Pantoea agglomerans, *and *Sphingomonas paucimobilis*.

### 3.3. Cell Phones

From 40 cell phones sampled, a total of 58 isolations were obtained, from those, 51 (88%) were considered potentially clinically relevant (Table 3), highlighting bacteria as MRSH, MRSC, MRSE, Methicillin-resistant *S. hominis* (MRSh)*, Pantoea agglomerans, Acinetobacter lwoffii *and *Sphingomonas paucimobilis.*

### 3.4. White Coats

From 35 with coats sampled, a total of 52 isolations were obtained, from those, 39 (75%) were considered potentially clinically relevant (Table 4), highlighting bacteria as *Pseudomonas oryzihabitans, *MRSE, MRSH, MRSh, and *Moraxella *sp.

### 3.5. Ties

From 22 ties sampled, a total of 27 isolations were obtained, from those, 18 (66.6%) were considered potentially clinically relevant (Table 5), highlighting bacteria as Methicillin resistant *S. aureus *and MRSE.

## 4. Discussion

The prevalence of antibiotic resistant bacteria is a serious problem with important implications for hospital infection control. Some studies have found bacterial contamination in the community (of cell phones) to be nearly equivalent to hospital settings [15]. Yet antibiotic resistant bacteria remain more common in hospital settings. Although the geographic distribution of these bacteria is worldwide, the epidemiology and dissemination patterns appear to differ within and across regions [1–8]. In this study, we found an alarming number of potentially clinically relevant bacteria colonizing different surfaces, these bacterial reservoirs are a plausible source of infection for patients at this tertiary level hospital and likely any other hospital worldwide.

The most important implication of our study is to highlight the role of these items as bacterial reservoirs and how HCWs should perform hand hygiene after contact with any clothes or environmental item in agreement with the recommendation of the guideline on hand hygiene in healthcare settings [1, 3]. Some other strategies to reduce the potential for transmission of pathogens from this surfaces include improved or more frequent cleaning [4, 6–8].

In contrast to previous studies on the role of environmental colonization that were performed during nosocomial pathogen outbreaks [21, 24], our study was conducted when there was no outbreak and reflects the regular daily risk of colonization or infection from hospital fomites. Bacterial contamination of items in health care settings is likely ongoing as organisms such as *Staphylococci, E. coli,* and *P. aeruginosa *survive at least 3–6 months on dried blood or cotton and as long as four weeks on other surfaces [26, 27]. Unfortunately, we did not investigate other factors in the transmission route, such as HCWs' hand carriage and colonization of patients.

## 5. Conclusion

This hospital-based cross-sectional surveillance study demonstrates that a large proportion of health care workers' clothing and personal effects were contaminated with bacterial pathogens that can result in nosocomial infections. Further research is needed to evaluate strategies to minimize the risk of patient-to-patient transmission of pathogens from other contaminated items.

## Figures and Tables

**Table 1:** Distribution of bacterial isolates from keyboards.

			Potentially clinically relevant microorganisms								Potentially clinically irrelevant microorganisms	

Type of surface	Number of samples per hospital area	Number of Isolates	Meticillin-resistant *Staphylococcus *sp.*		Meticillin-sensible *Staphylococcus *sp.^+^		*Enterococcus *sp.*^^^*		Gram-negative rods^†^		*Bacillus* sp.	
			*n*	%	*n*	%	*n*	*%*	*n*	%	*n*	%
Keyboards (*n* = 30)	Emergency room (*n* = 7)	8	0	0	3	37,5	0	0	2	25	3	37,5
	Adult surgical ICU (*n* = 6)	8	0	0	6	75	2	25	0	0	0	0
	Adult medical ICU (*n* = 6)	9	0	0	3	33,3	1	11,1	1	11,1	4	44,4
	Adult special care unit (*n* = 6)	9	1	11,1	2	22,2	0	0	0	0	6	66,6
	Internal medicine ward (*n* = 5)	5	1	20	0	0	0	0	0	0	4	80

	Overall	**39**	**2**	**5,1**	**14**	**35,9**	**3**	**7,7**	**3**	**7,7**	**17**	**43,5**

*S. epidermidis. ^+^ *S. epidermidis, S. aureus, S. warneri, S. haemolyticus. ^^^*One was a *E. faecium* resistant to vancomycin. ^†^ *Pantoea agglomerans, Escherichia hermannii, Leclercia adecarboxylata.*

**Table 2:** Distribution of bacterial isolates from curtains.

			Potentially clinically relevant microorganisms						Potentially clinically irrelevant microorganisms	

Type of surface	Number of samples per Hospital area	Number of Isolates	Meticillin-resistant *Staphylococcus *sp.*		Meticillin-sensible *Staphylococcus *sp.^+^		Gram-negative rods^†^		*Bacillus* sp.	
			*n*	%	*n*	%	*n*	%	*n*	%
Curtains (*n* = 32)	Emergency room (*n* = 8)	13	4	30,8	6	46,1	1	7,7	2	15,3
	Adult surgical ICU (*n* = 5)	8	4	50	3	37,5	0	0	1	12,5
	Adult medical ICU (*n* = 2)	2	1	50	1	50	0	0	0	0
	Adult special care unit (*n* = 6)	14	3	21,4	4	28,5	1	7,1	6	42,8
	Internal medicine ward (*n* = 11)	22	6	27,2	7	31,8	6	27,2	3	13,6

	Overall	59	18	30,5	21	35,5	8	13,5	12	20,3

*S. epidermidis, S. haemolyticus, S. cohnii, S. saprophyticus, S. hominis. ^+^ *S. epidermidis, S. aureus, S. cohnii, S. hominis, S. haemolyticus, S. warneri, S. sciuri, S. saprophyticus.* ^†^ *Acinetobacter ursingii, Pantoea agglomerans, Moraxella *sp.*, Pseudomonas oryzihabitans *AMP-C producer, *Sphingomonas paucimobilis, Pasteurella multocida.*

**Table 3:** Distribution of bacterial isolates from cell phones.

			Potentially clinically relevant microorganisms						Potentially clinically irrelevant microorganisms	

Type of surface	Number of samples per doctor specialty	Number of Isolates	Meticillin-resistant *Staphylococcus *sp.*		Meticillin-sensible *Staphylococcus *sp.^+^		Gram-negative rods^†^		*Bacillus* sp.	
			*n*	%	*n*	%	*n*	%	*n*	%
Cell phones (*n* = 40)	General (*n* = 7)	11	2	18,1	8	72,7	1	9,1	0	0
	Internal medicine (*n* = 8)	9	2	22,2	5	55,5	1	11,1	1	11,1
	Clinical resident (*n* = 6)	9	3	33,3	4	44,4	1	11,1	1	11,1
	Surgery (*n* = 3)	6	3	50	3	50	0	0	0	0
	Surgery resident (*n* = 4)	4	1	25	2	50	0	0	1	25
	Medical student (*n* = 9)	15	0	0	9	60	2	13,3	4	26,6
	Nurse (*n* = 1)	**1**	**0**	0	**1**	100	**0**	0	**0**	0
	Nutritionist (*n* = 2)	**3**	**0**	0	**3**	100	**0**	0	**0**	0

	Overall	**58**	**11**	19	**35**	60,3	**5**	8,6	**7**	12

*S. epidermidis, S. cohnii, S. hominis, S. haemolyticus. ^+^ *S. epidermidis, S. aureus, S. haemolyticus, S. hominis, S. warneri, S. chromogenes.* ^†^ *Acinetobacterlwoffii, Pantoea agglomerans, Aeromonas salmonicida, Sphingomonas paucimobilis.*

**Table 4:** Distribution of bacterial isolates from white coats.

			Potentially clinically relevant microorganisms						Potentially clinically irrelevant microorganisms	

Type of surface	Number of samples per doctor specialty	Number of Isolates	Meticillin-resistant *Staphylococcus *sp.*		Meticillin-sensible *Staphylococcus *sp.^+^		Gram-negative rods^†^		*Bacillus* sp.	
			*n*	%	*n*	%	*n*	%	*n*	%
White coats (*n* = 35)	General (*n* = 4)	9	0	0	5	55,5	1	11,1	3	33,3
	Internal medicine (*n* = 12)	19	1	5,2	11	57,9	1	5,2	6	31,5
	Clinical resident (*n* = 7)	7	2	28,5	4	57	0	0	1	14,2
	Surgery (*n* = 5)	7	1	14,2	5	71	0	0	1	14,2
	Surgery resident (*n* = 4)	5	1	20	2	40	2	40	0	0
	Medical student (*n* = 2)	3	0	0	2	66,6	0	0	1	33,3
	Nutritionist (*n* = 1)	**2**	0	0	1	50	0	0	**1**	50

	Overall	**52**	**5**	9,6	**30**	57,7	**4**	7,7	**13**	25

*S. epidermidis, S. haemolyticus. S. hominis. ^+^ *S. capitis, S. aureus, S. warneri, S. epidermidis.* ^†^ *Pseudomonas oryzihabitans *AMP-C producer, *Moraxella* sp.

**Table 5:** Distribution of bacterial isolates from ties.

			Potentially clinically relevant microorganisms				Potentially clinically irrelevant microorganisms	

Type of surface	Number of samples per doctor specialty	Number of Isolates	Meticillin-resistant *Staphylococcus *sp.*		Meticillin-sensible *Staphylococcus *sp.^+^		*Bacillus* sp.	
			*n*	%	*n*	%	*n*	%
Ties (*n* = 22)	General (*n* = 4)	6	0	0	4	66,6	2	33,3
	Internal medicine (*n* = 10)	13	2	15,3	6	46,1	5	38,4
	Surgery (*n* = 6)	6	1	16,6	4	66,6	1	16,6
	Medical student (*n* = 2)	2	0	0	1	50	1	50

	Overall	**27**	**3**	11,1	**15**	55,5	**9**	33,3

*S. aureus, S. epidermidis. ^+^ *S. epidermidis, S. hominis, S. warneri, S. aureus.*

## References

[1] Boyce JM, Pittet D (2002). Guideline for hand hygiene in health-care settings: recommendations of the Healthcare Infection Control Practices Advisory
Committee and the HICPAC/SHEA/APIC/IDSA Hand Hygiene Task Force. *Infection Control and Hospital Epidemiology*.

[2] Bhalla A, Pultz NJ, Gries DM (2004). Acquisition of nosocomial pathogens on hands after contact with environmental surfaces near hospitalized patients. *Infection Control and Hospital Epidemiology*.

[3] Boyce JM, Potter-Bynoe G, Chenevert C, King T (1997). Environmental contamination due to methicillin-resistant Staphylococcus aureus: possible infection control implications. *Infection Control and Hospital Epidemiology*.

[4] Hayden MK, Bonten MJM, Blom DW, Lyle EA, Van De Vijver DAMC, Weinstein RA (2006). Reduction in acquisition of vancomycin-resistant *Enterococcus* after enforcement of routine environmental cleaning measures. *Clinical Infectious Diseases*.

[5] Ray AJ, Hoyen CK, Taub TF, Eckstein EC, Donskey CJ (2002). Nosocomial transmission of vancomycin-resistant enterococci from surfaces. *Journal of the American Medical Association*.

[6] Denton M, Wilcox MH, Parnell P (2004). Role of environmental cleaning in controlling an outbreak of Acinetobacter baumannii on a neurosurgical intensive care unit. *Journal of Hospital Infection*.

[7] Mayfield JL, Leet T, Miller J, Mundy LM (2000). Environmental control to reduce transmission of Clostridium difficile. *Clinical Infectious Diseases*.

[8] Wilcox MH, Fawley WN, Wigglesworth N, Parnell P, Verity P, Freeman J (2004). Comparison of the effect of detergent versus hypochlorite cleaning on
environmental contamination and incidence of Clostridium difficile infection. *Journal of Hospital Infection*.

[9] Das I, Lambert P, Hill D, Noy M, Bion J, Elliott T (2002). Carbapenem-resistant Acinetobacter and role of curtains in an outbreak in intensive care units. *Journal of Hospital Infection*.

[10] Klakus J, Vaughan NL, Boswell TC (2008). Meticillin-resistant Staphylococcus aureus contamination of hospital curtains. *Journal of Hospital Infection*.

[11] Trillis F, Eckstein EC, Budavich R, Pultz MJ, Donskey CJ (2008). Contamination of hospital curtains with healthcare-associated pathogens. *Infection Control and Hospital Epidemiology*.

[12] Kilic IH, Ozaslan M, Karagoz ID, Zer Y, Davutoglu V (2009). The microbial colonization of mobile phone used by heatlh care staff. *Pakistan Journal of Biological Sciences*.

[13] Brady RRW, Verran J, Damani NN, Gibb AP (2009). Review of mobile communication devices as potential reservoirs of nosocomial pathogens. *Journal of Hospital Infection*.

[14] Ulger F, Esen S, Dilek A, Yanik K, Gunaydin M, Leblebicioglu H (2009). Are we aware how contaminated our mobile phones with nosocomial pathogens?. *Annals of Clinical Microbiology and Antimicrobials*.

[15] Akinyemi KO, Atapu AD, Adetona OO, Coker AO (2009). The potential role of mobile phones in the spread of bacterial infections. *Journal of Infection in Developing Countries*.

[16] Lopez PJ, Ron O, Parthasarathy P, Soothill J, Spitz L (2009). Bacterial counts from hospital doctors’ ties are higher than those from shirts. *American Journal of Infection Control*.

[17] Treakle AM, Thom KA, Furuno JP, Strauss SM, Harris AD, Perencevich EN (2009). Bacterial contamination of health care workers’ white coats. *American Journal of Infection Control*.

[18] Snyder GM, Thom KA, Furuno JP, Perencevich EN, Roghmann MC, Strauss SM (2008). Detection of methicillin-resistant staphylococcus aureusand vancomycin-resistant enterococci on the gowns and gloves of healthcare workers. *Infection Control and Hospital Epidemiology*.

[19] Fukada T, Iwakiri H, Ozaki M (2008). Anaesthetists’ role in computer keyboard contamination in an operating room. *Journal of Hospital Infection*.

[20] Lu PL, Siu LK, Chen T-C (2009). Methicillin-resistant Staphylococcus aureus and Acinetobacter baumannii on computer interface surfaces of hospital wards and association with clinical isolates. *BMC Infectious Diseases*.

[21] Hartmann B, Benson M, Junger A (2004). Computer keyboard and mouse as a reservoir of pathogens in an intensive care unit. *Journal of Clinical Monitoring and Computing*.

[22] Schultz M, Gill J, Zubairi S, Huber R, Gordin F (2003). Bacterial contamination of computer keyboards in a teaching hospital. *Infection Control and Hospital Epidemiology*.

[23] Boyce JM (2007). Environmental contamination makes an important contribution to hospital infection. *Journal of Hospital Infection*.

[24] Burden M, Cervantes L, Weed D, Keniston A, Price CS, Albert RK (2011). Newly cleaned physician uniforms and infrequently washed white coats have similar rates of bacterial contamination after an 8-hour workday: a randomized controlled trial. *Journal of Hospital Medicine*.

[25] Pandey A, Asthana AK, Tiwari R, Kumar L, Das A, Madan M (2010). Physician accessories: doctor, what you carry is every patient’s worry. *Indian Journal of Pathology and Microbiology*.

[26] Smith SM, Eng RHK, Padberg FT (1996). Survival of nosocomial pathogenic bacteria at ambient temperature. *Journal of Medicine*.

[27] Harris AD (2008). How important is the environment in the emergence of nosocomial antimicrobial-resistant bacteria?. *Clinical Infectious Diseases*.

